# FSH enhances the inflammatory response of macrophages in the knee joint possibly through the NFκB pathway

**DOI:** 10.1002/2211-5463.13959

**Published:** 2025-01-13

**Authors:** Yu Chen, Na Xu, Wen‐wen Zhang, Yan Wang, Tong Su, Yan‐man Zhou, Jin Xu

**Affiliations:** ^1^ Key Laboratory of Endocrine Glucose & Lipids Metabolism and Brain Aging Ministry of Education Jinan China; ^2^ Department of Endocrinology Shandong Provincial Hospital Affiliated to Shandong First Medical University Jinan China; ^3^ Shandong Key Laboratory of Endocrinology and Lipid Metabolism Jinan China; ^4^ Shandong Institute of Endocrine and Metabolic Diseases Jinan China; ^5^ “Chuangxin China” Innovation Base of Stem Cell and Gene Therapy for Endocrine Metabolic Diseases Jinan China; ^6^ Shandong Engineering Laboratory of Prevention and Control for Endocrine and Metabolic Diseases Jinan China; ^7^ Shandong Engineering Research Center of Stem Cell and Gene Therapy for Endocrine and Metabolic Diseases Jinan China; ^8^ Department of Nephrology Shandong Provincial Hospital Affiliated to Shandong First Medical University Jinan China

**Keywords:** FSH, macrophages, NFκB, osteoarthritis, TNF‐α

## Abstract

Previous studies have suggested that women with higher follicle‐stimulating hormone (FSH) levels have a greater incidence of osteoarthritis (OA) compared to women with lower FSH despite normal estrogen levels. Our previous studies also showed that FSH has a negative effect on cartilage in postmenopausal OA. However, no studies have investigated the effect of FSH on the synovium. Here, we showed that the FSH receptor (FSHR) is expressed on RAW264.7 cells and BMDM (Bone Marrow‐Derived Macrophages), and found that FSH stimulation promotes the production and secretion of inflammatory cytokines in synovial macrophages. In RAW264.7 cells, FSH stimulation enhances phosphorylation and nuclear translocation of P65, suggesting the activation of NFκB signaling, while the knockdown of FSHR eliminates the proinflammatory effect of FSH. To further validate these results, we used an ovariectomy mouse model supplemented with FSH and estrogen, and a mouse model with FSH neutralization. We noted that FSHR was expressed on mouse synovial joint membranes. Furthermore, in ovariectomy mice supplemented with estrogen and treated with FSH, synovial macrophages were significantly increased, while the opposite was the case in the FSH neutralizing group, which suggest that FSH triggers an inflammatory response in the synovial tissue in mice. Taken together, our results indicate that FSH is an important regulator in synovial inflammation via NFκB signaling activation and, to some extent, appears to accelerate the development of osteoarthritis.

## Graphical abstract

We explored the role of FSH on the synovium and found that FSH was able to mediate the development of osteoarthritis by acting on the FSH receptor (FSHR) to promote the production and release of inflammatory factors through the activation of the NFκB signaling pathway.

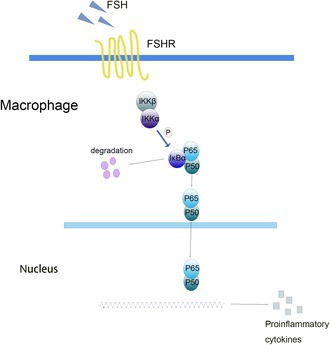

**Abbreviations**
BMDM: bone marrow‐derived macrophages
CCL11: chemokine ligand 11
CCL5: chemokine (C‐C motif) ligand 5
DMEM: Dulbecco's modified eagle medium
DMSO: dimethyl sulfoxide
ECM: extracellular matrix
FBS: Fetal bovine serum
FSH: follicle stimulating hormone
FSHAb: follicle stimulating hormone antibody
FSHR: follicle stimulating hormone receptor
GM‐CSF: granulocyte‐macrophage colony‐stimulating factor
H&E: hematoxylin–eosin staining
IFN‐γ: interferon‐γ
IgG: immunoglobulin G
IKKβ: inhibitor of kappa B kinase β
IL‐10: interleukin10
IL‐12(P40): interleukin12(P40)
IL‐12(P70): interleukin12(P70)
IL‐1a: interleukin1a
IL‐1b: interleukin1b
IL‐2: interleukin2
IL‐5: interleukin5
IL‐6: interleukin6
IL‐9: interleukin9
KOA: knee osteoarthritis
MMP: matrix metalloproteinase
MOST: multicenter osteoarthritis study
NFκB: nuclear factor kappa‐B
OA: osteoarthritis
PVDF: polyvinyligene fluoride
qRT‐PCR: reverse transcription‐polymerase chain reaction
RA: rheumatic arthritis
TNF‐α: tumor necrosis factor α

Osteoarthritis (OA) is a major cause of joint pain, disability, and healthcare costs worldwide [1], and involves the whole joint (including cartilage, synovium, and subchondral bone). Cartilage degeneration, bone formation, and synovial inflammation are always observed in OA. Among them, synovium is a specialized connective tissue arranged around the joints, surrounding the tendons and forming the lining of the capsule and fat pads, which secretes synovial fluid and protects the articular cartilage. Synovial inflammation follows the entire course of osteoarthritis; traditionally, this has been primarily thought of as an associated disease due to joint overload or overuse, but there is growing evidence that synovitis and the resulting proinflammatory mediators are important in the pathogenesis of OA, with implications for articular cartilage [2, 3], and a multicenter osteoarthritis study (MOST) found synovitis to be an independent risk factor for knee OA [4]. Therefore, synovium also plays an important role in OA.

Studies have shown that there is a significant increase in the prevalence of OA in postmenopausal women [5, 6], and estrogen plays an important role in it [1]; however, estrogen is not the only regulator that affects OA. Previous studies found that there was an elevated follicle stimulating hormone (FSH) levels in postmenopausal women with KOA (knee osteoarthritis), but their estrogen levels are normal [7, 8], suggesting that FSH may play a role in the development of postmenopausal OA.

FSH regulates physiological processes by blinding to FSHR. FSHR belongs to a group of seven transmembrane G‐protein‐coupled receptors that are mainly expressed in the gonads. However, FSHR has also been found to be expressed elsewhere [9] (e.g. tumors, monocytes, osteoblasts, and chondrocytes) and the role of FSH in osteoclasts and chondrocytes has been verified [10, 11]. However, whether FSHR is expressed on the synovium has never been explored.

As mentioned above, synovium is a specialized connective tissue that plays an important role in OA [12]. The synovial inflammation and inflammatory mediators are important in the pathogenesis of OA [2, 13]; in particular, inflammatory mediators released by the synovium can promote synovial inflammation and synovial inflammation directly contributes to several clinical signs and symptoms, including joint swelling and effusion, and reflects the structural progression of the disease. Previous studies have shown that macrophages are the predominant immune cells in synovium [14], which respond to OA by producing inflammatory mediators that in turn attract immune cells and induce phenotypic transformation of chondrocytes [15]. Meanwhile, our previous studies have shown that FSH exerted a proinflammatory role on OA cartilage; one study also found higher FSH levels in OA synovial fluid [16]. Therefore, it is important to study the inflammatory role of FSH in synovial macrophages.

## Materials and methods

### Animal model

C57BL/6J female mice purchased from Charles River (Beijing, China) were used in two animal models. In an animal model, the mice were divided into three groups: sham, OVX, OVX + E2 + FSH, sham group only operated with skin incision and suturing, as the negative control; the OVX group was treated with ovariectomy, as the positive control based on the confirmation that estrogen deficiency caused postmenopausal osteoarthritis [17, 18]; the OVX + E2 + FSH group was supplemented with E2 chow to eliminate the effects of estrogen deficiency in ovariectomy mice, and was given 30 IU/(KG, d) FSH (Geneva, Merckserono, Switzerland) for 2 weeks, to observe the independent effects of FSH on joints. In the other animal model, the mice were divided into two groups: IgG and FSHAb groups. FSHAb and IgG groups were separately given anti‐FSHβ antibodies (Medix Mab, #6602, BiosPacific, Emeryville, CA, USA), and anti‐IgG antibody (Proteintech, Wuhan, China) at 100 μg·day^−1^ for 8 weeks. The same group was kept in the same cages and all groups were kept in the same environment.

The mice used in the studies were bled to death after intraperitoneal injection with 240 mg·kg^−1^ of tribromoethanol. All procedures in studies were in accordance with the ethical standards of the Shandong Provincial Hospital affiliated with Shandong First Medical University (SWYX: NO.2022–036).

### Western blot

RAW264.7 cells were lysed with Cell Lysis Buffer with protease inhibitors. Proteins were electrophoresed on SDS gels and transferred to PVDF membranes, sealed with 5% skim milk; nucleus and cytoplasmic proteins were extracted by the Nucleus and Cytoplasmic Protein Extraction Kit (Beyotime, Shanghai, China). Proteins were incubated with antibodies against P65 (1:1000 CST, Danvers, MA, USA), p‐P65 (1:1000 CST, Danvers, MA, USA), IKKβ (1:1000 CST, Danvers, MA, USA), GAPDH (1:1000 CST, Danvers, MA, USA), IL‐10 (1:1000 Abcam, Cambridge, Cambridgeshire, UK), actin (1:7500 Abclonal, Woburn, MA, USA) by using secondary antibodies in combination with primary antibodies. The membrane‐bound antibodies were detected using the Immobilon Western chemiluminescent HRP substrate (Millipore, Billerica, MA, USA).

### Quantitative real‐time PCR (qRT‐PCR)

After extracting RNA with TRIZOL, the RNA (1000 ng) was transcribed into cDNA, and subjected to qRT‐PCR in a Roche LightCycler480 system by using the following cycle conditions: 95 °C‐5 min, 95 °C‐10 s, 60 °C‐10 s, 72 °C‐10 s, for 45 cycles. (The primer's sequence is shown in Table S1).

### Luminex

Luminex (ThermoFisher, Waltham, MA, USA) liquid‐phase microarray technology was used to detect the RAW264.7 cell culture supernatant. After centrifugation, the Luminex test was carried out according to the instructions of Luminex.

### Immunohistochemistry

After dehydration, antigen repair, blocking, and membrane permeabilization was added to BSA to seal paraffin sections. Used the diluted F4/80 antibody (1:500, Wuhan Seville, Wuhan, China), FSHR (1:500, Protein Tech, Rosemont, IL, USA), TNF‐α (1:100, Abcam) overnight. The next day, we added a secondary antibody to incubate, colored it with DAB, then redyed and observed after dehydration.

### Immunofluorescence

The cells fixed with paraformaldehyde, infiltrated with Triton X‐100, and then sealed with BSA. The F4/80 (1:500, Wuhan Servicebio), FSHR (1:500, Protein Tech), P65 (1:1000, CST), TNF‐α (1:100, Abcam), IL‐6 (1:500, Protein Tech) were cultured overnight. The secondary antibody (1:1000, Invitrogen, Carlsbad, CA, USA) were incubated and kept away from light, then sealed with DAPI containing blocker (Abcam). Image capture used a laser confocal microscope (Leica, Weitzlar, Germany).

### CCK8

The cells were seeded in 96‐well plates at a density of 8 × 10^3^ cells per well, and treated with different concentrations of FSH (0, 25, 50, 100 ng·mL^−1^), and added with 10 μL per well of CCK8 for the test at 24, 48, 72 h. Detection was at a wavelength of 450 nm.

### Histology

The paraffin sections of the leg bones were sectioned, and the paraffin sections were sectioned by H&E staining and Safranin O staining.

### Cell isolation and culture

RAW264.7 are murine macrophage cells that are widely used in synovial macrophages studies [19, 20]. The RAW264.7 cell was purchased from ATCC Cell Bank (Shanghai, China), cell catalog number ATCC TIB‐71, and cultured with 10% FBS and 90% DMEM high‐glucose medium.

BMDM was extracted from mouse bone marrow, lysed by erythrocytes, and placed in 10% FBS, 1% penicillin/streptomycin, and DMEM high‐glucose medium overnight, and on the following day the suspended cells were taken and induced to differentiate into BMDM with medium containing 10% FBS, 1% penicillin/streptomycin, 35 ng·mL^−1^ M‐CSF, and DMEM high‐glucose medium.

### FSH stimulation

FSH was purchased from R&D System (Minneapolis, MN, USA). After starving for 2 h, the RAW264.7 cells and BMDM were directly stimulated by FSH.

### Transfection of siRNA

Three pairs of siRNA (si‐1#, si‐2#, si‐3#) (OBiO, China, Shanghai) were used to transfect the RAW264.7 cell line with lipo3000 (Invitrogen, La Jolla, CA, USA). The primer sequences are shown in Table S2.

### ELISA assay

The supernatant of RAW264.7 cells was collected, and then used according to the ELISA assay protocol, TNF‐α, Estrogen (Cusabio, Wuhan, China), FSH (Abnova, Taipei, Taiwan). The OD value was measured at 450 nm.

### Statistical analysis

The difference between the two groups was determined by a *t*‐test, and the difference among more than or equal to the three groups was determined by one‐way ANOVA and two‐way ANOVA. All immunofluorescence fluorescence intensity quantifications were analyzed using image j software (National Institutes Of Health (NIH), Bethesda, MD, USA). All data analyses were performed using graphpad prism 9 (GRAPHPAD Software, San Diego, CA, USA). *P* < 0.05 were considered as statistical difference. Quantitative data are presented as mean ± SD.

## Results

### FSH stimulation promoted the production of inflammatory cytokines in macrophages

**Fig. 1:**
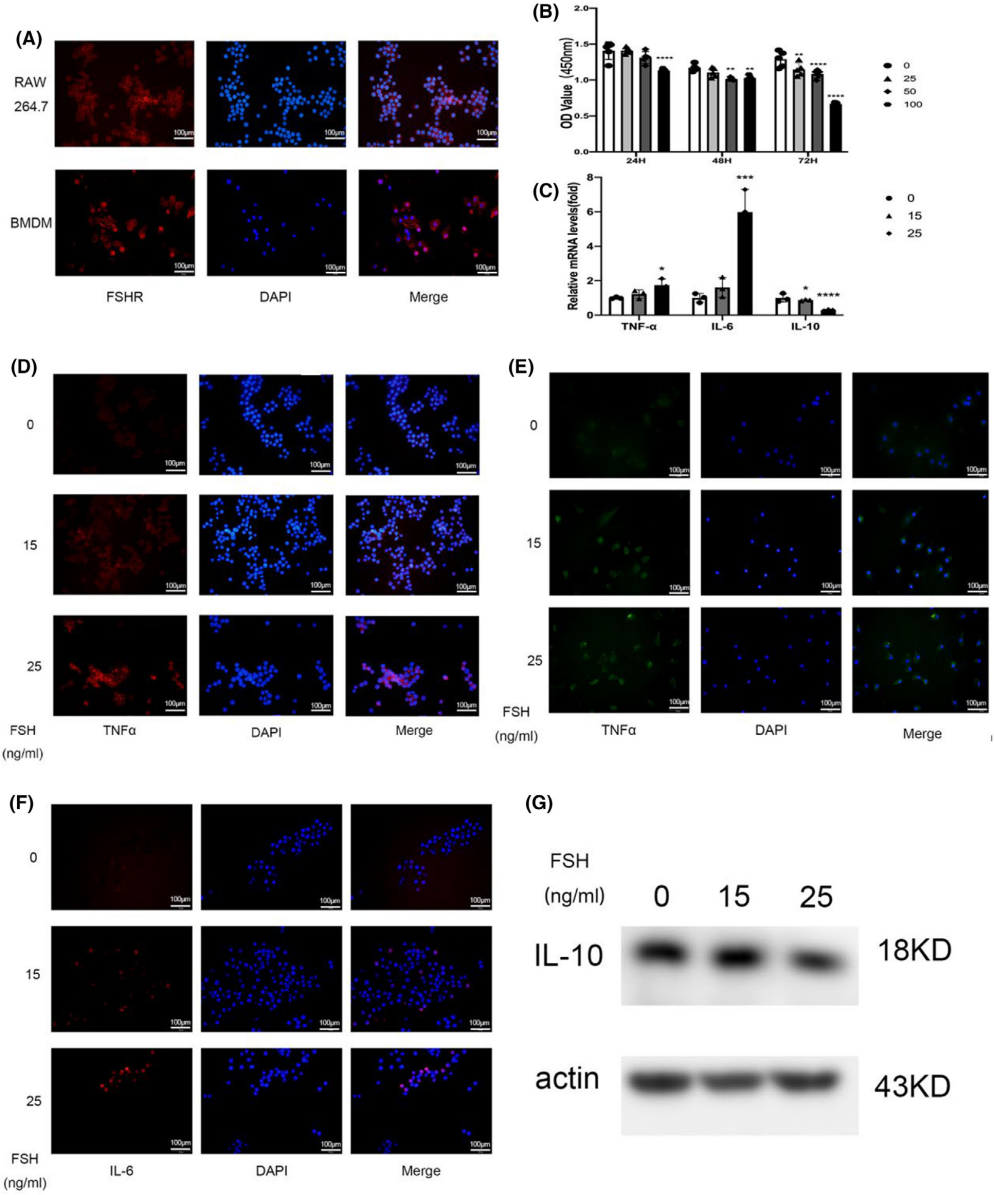
FSH stimulation promoted the production of inflammatory cytokines in macrophages. (A) The localization of FSHR was visualized by immunofluorescence in both RAW264.7 cells and BMDM; red indicates FSHR, cell nucleus stained with DAPI (blue). (B) CCK‐8 assay showed the effect of FSH on the viability of RAW264.7 cells, *n* = 5; data are presented as means ± SD, data were analyzed by two‐way ANOVA, **P < 0.01, ****P < 0.0001 relative to control. (C) RAW264.7 cells were stimulated with FSH (0, 15, 25 ng·mL^−1^); the mRNA expression of TNF‐α, IL‐6, IL‐10 was detected by qRT‐PCR, *n* = 3; data are presented as means ± SD, data were analyzed by two‐way ANOVA, *P < 0.05, ***P < 0.001, ****P < 0.0001 relative to control. (D) After FSH stimulation (0, 15, 25 ng·mL^−1^), the protein level of TNF‐α was detected by immunofluorescence in RAW264.7 cells. Red and green indicate TNF‐α, blue indicates nuclei stained with DAPI. (E) After FSH stimulation (0, 15, 25 ng·mL^−1^), the protein level of TNF‐α was detected by immunofluorescence in BMDM. Red and green indicate TNF‐α, blue indicates nuclei stained with DAPI. (F) RAW264.7 cells were stimulated with FSH (0, 15, 25 ng·mL^−1^), IL‐6 was detected by immunofluorescence at the protein level. Red indicates IL‐6, cell nucleus stained with DAPI (blue). Scale bar = 100 μm. (G) RAW264.7 cells were stimulated with FSH (0, 15, 25 ng·mL^−1^), IL‐10 was detected by western blot at the protein level, *n* = 3.

Our results suggested that FSHR was expressed on RAW264.7 cells and BMDM (Fig. 1A) by the method of immunofluorescence. Then we detected the viability of RAW264.7 cells after FSH stimulation by CCK8 assay, and found that the short‐time (< 24 h) and low‐concentration (< 50 ng·mL^−1^) of FSH stimulation had no effect on macrophage viability, but long‐time (> 24 h) and high‐concentration (>50 ng·mL^−1^) stimulation decreased cell viability (Fig. 1B). Therefore, short‐time and low‐concentration FSH stimulation was used in all subsequent experiments.

**Fig. 2:**
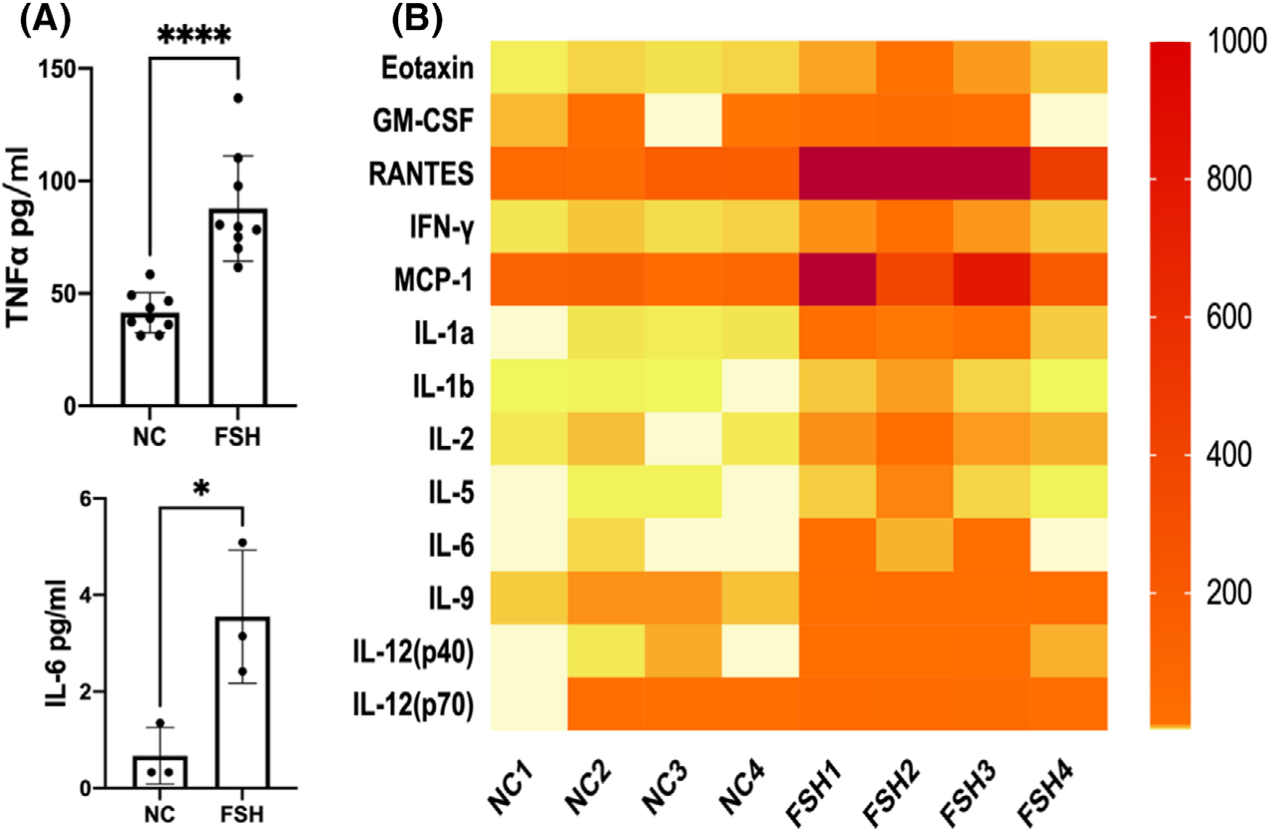
FSH stimulation increased secretion of different inflammatory cytokines. (A) RAW264.7 cells were stimulated by 25 ng·mL^−1^ FSH, the level of TNF‐α in the supernatant was detected by ELISA, the level of IL‐6 was detected by Luminex, *n* = 9, data are presented as means ± SD, data were analyzed by *t*‐test, *P < 0.05, ****P < 0.0001 relative to NC. (B) RAW264.7 cells were stimulated by 25 ng·mL^−1^ FSH. A variety of inflammatory cytokines were significantly increased in the supernatant detected by Luminex. The red area indicates high levels of inflammatory cytokines, while the white area indicates the data lower than detected.

We assessed the intracellular production of inflammatory cytokines in macrophages (RAW264.7 and BMDM) after FSH stimulation (Fig. 1C–F and Fig. S1), and found that FSH stimulation increased the production of inflammatory cytokines (IL‐6, TNF‐α), while reducing the production of antiinflammatory cytokine IL‐10 in a dose‐dependent manner. We also assessed the secretion of TNF‐α and IL‐6 (Fig. 2A), and these were significantly increased after FSH stimulation (25 ng·mL^−1^). To examine whether FSH could stimulate the production of other inflammation cytokines, we tested various inflammatory cytokines (Fig. 2B) by using Luminex, and found that other inflammatory cytokines, including IL‐1a, IL‐1b, IL‐2, IL‐9, eotaxin (CCL11), IFN‐γ, GM‐CSF, and RANTES (CCL5) were significantly elevated (Fig. S2) after FSH treatment. The above results indicated that FSH stimulation increased the production of inflammatory cytokines in macrophages.

### Knockdown of FSHR reversed the proinflammatory effect of FSH in macrophages

**Fig. 3:**
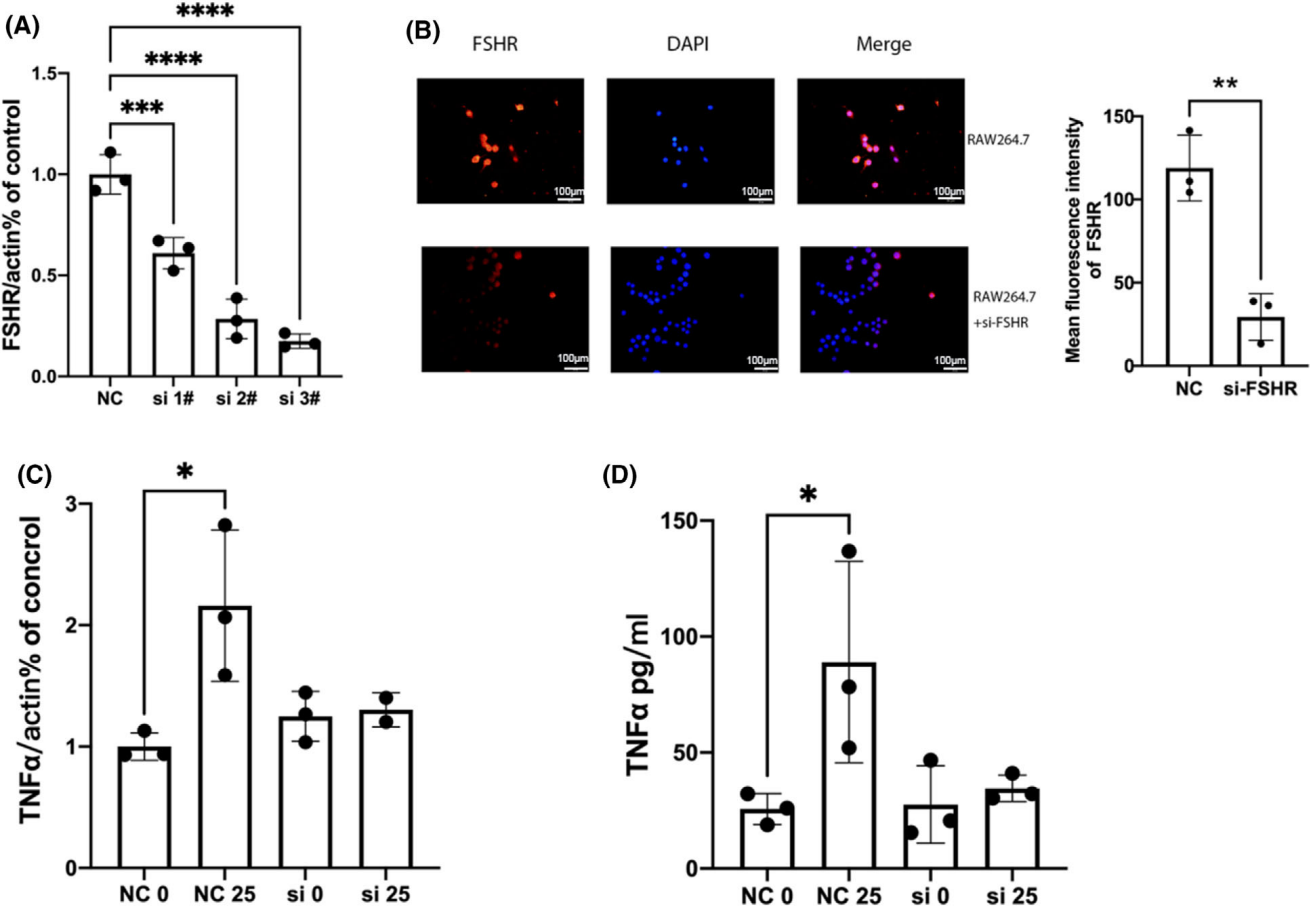
Knockdown of FSHR reversed the production of TNF‐α stimulated by FSH in macrophages. (A) The efficiency of FSHR knockdown was detected by qRT‐PCR in RAW264.7 cells, *n* = 3, data are presented as means ± SD; data were analyzed by one‐way ANOVA, ***P < 0.001, ****P < 0.0001 relative to NC. (B) The efficiency of FSHR knockdown by si‐3# was tested by immunofluorescence in RAW264.7 cells. Red indicates FSHR, cell nucleus stained with DAPI (blue), scale bar = 100 μm; data are presented as means ± SD, data were analyzed by *t*‐test, **P < 0.01. (C) The mRNA expression of TNF‐α was detected by qRT‐PCR in RAW264.7 cells; data are presented as means ± SD, data were analyzed by one‐way ANOVA, *P < 0.05 relative to NC0. (D) The protein level of TNF‐α were detected by ELISA in RAW264.7 cells; data are presented as means ± SD, data were analyzed by one‐way ANOVA, *P < 0.05 relative to NC0.

We knocked down the FSHR in macrophages by using siRNA and the knockdown efficiency was detected by qRT‐PCR (Fig. 3A), and showed that the third pair of siRNA (si‐3#) had the highest knockdown efficiency; then we further verified the protein knockdown efficiency of si‐3# by immunofluorescence (Fig. 3B). TNF‐α is a representative inflammatory cytokine in OA, so we tested the effect of FSHR knockdown on TNF‐α, and found that the knockdown of FSHR both inhibited the mRNA expression and secretion of TNF‐α stimulated by FSH (Fig. 3C,D), indicating that FSH played a proinflammatory role through FSHR.

### FSH stimulation activated the NFκB signaling pathway

**Fig. 4:**
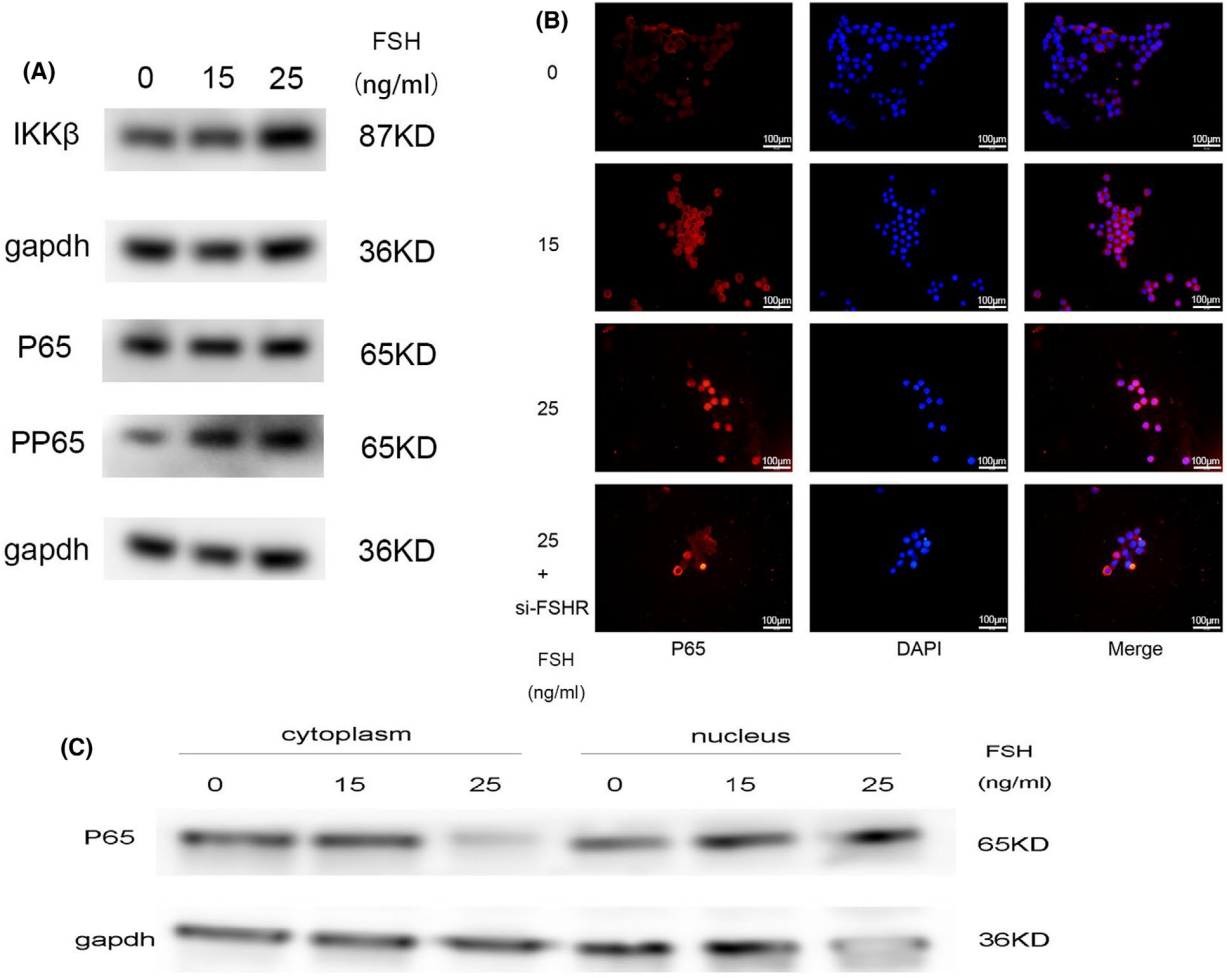
FSH stimulation activated the NFκB signaling pathway. (A) RAW264.7 cells were stimulated with FSH (0, 15, 25 ng·mL^−1^), and the protein levels of P65, P‐P65, and IKKβ were observed by western blot, *n* = 3. (B) The nucleation of P65 was visualized by immunofluorescence in RAW264.7 cells; the results showed that FSH stimulation increased the nucleation of P65, while knockdown FSHR eliminated this phenomenon; red indicates P65, cell nucleus stained with DAPI (blue), scale bar = 100 μm. (C) RAW264.7 cells were stimulated with FSH (0, 15, 25 ng·mL^−1^), and the P65 protein levels in cytoplasm and nucleus were observed by western blot, *n* = 3.

The NFκB signaling pathway is a classical inflammatory pathway [21] that can be activated by phosphorylation and nucleation of P65, and it has been found to be involved in OA [22, 23]. IKKβ regulating the NFκB signaling pathway activation are very important. We found the protein level of IKKβ was elevated after FSH stimulation in a dose‐dependent manner (Fig. 4A). Then we examined the phosphorylation of P65 and found FSH significantly enhanced the phosphorylation of p65 in a dose‐dependent manner (Fig. 4A). We further found that the nucleation of p65 was also increased after FSH stimulation (Fig. 4B,C), which was suppressed by blocking FSHR (Fig. 4B). Above all, we speculated that the increase of inflammatory cytokines caused by FSH was mediated by NFκB signaling pathways.

### FSH triggered inflammation of the synovial tissue in mice

**Fig. 5:**
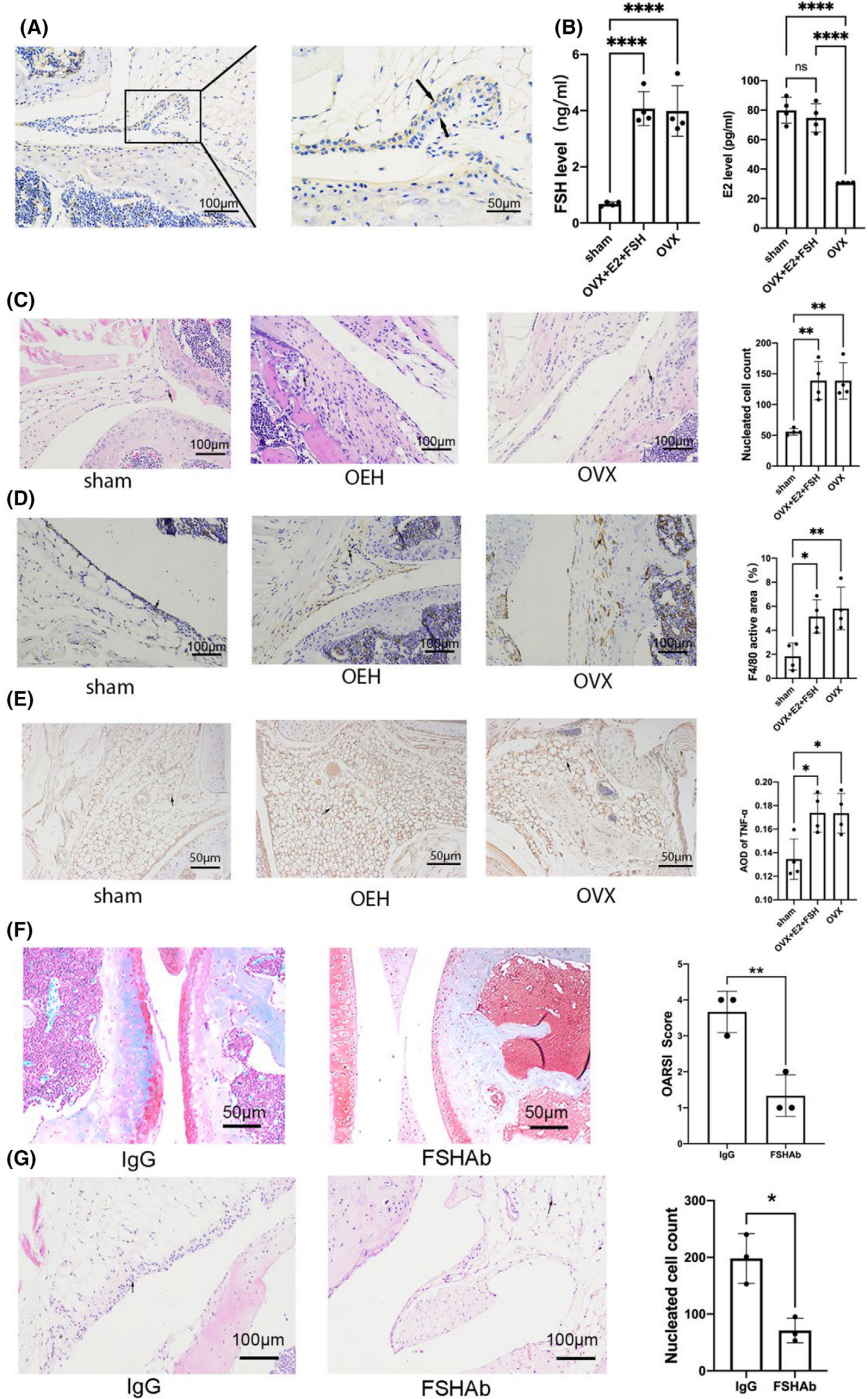
FSH triggered inflammation of synovial tissue of the knee in mice. (A) Immunostaining of FSHR in the synovial membrane of mouse knee joint, *n* = 4, scale bar = 100 μm (left panel), scale bar = 50 μm (right panel). (B) The levels of serum FSH and estrogen were detected by ELISA, *n* = 4; data are presented as means ± SD, data were analyzed by one‐way ANOVA, *P < 0.05, ***P < 0.001 relative to sham. (C) H&E staining of synovial membrane showed inflammatory cells in the OVX + E2 + FSH and OVX groups were increased compared to the sham group, *n* = 4; data are presented as means ± SD, data were analyzed by one‐way ANOVA, scale bar = 100 μm. (D) Macrophages were labeled by F4/80 immunostaining, and macrophages in the OVX + E2 + FSH and OVX groups were significantly increased in control compared to the sham group; *n* = 4, data are presented as means ± SD, data were analyzed by one‐way ANOVA, scale bar = 100 μm. (E) Synovial membrane was labeled by TNF‐α immunostaining, and TNF‐α in the OVX + E2 + FSH and OVX groups were significantly increased in control compared to the sham group, *n* = 4; data are presented as means ± SD, data were analyzed by one‐way ANOVA, scale bar = 100 μm. (F) Safranin O (red) and Fast Green staining of cartilage showed that cartilage destruction relief in the FSHAb group compared to the IgG group in mice, *n* = 3; data are presented as means ± SD, data were analyzed by *t*‐test, scale bar = 50 μm. (G) H&E staining of synovial membrane showed inflammatory cells in the FSHAb group decreased compared to the IgG group, *n* = 3; data are presented as means ± SD, data were analyzed by *t*‐test, scale bar = 100 μm.

We further verified the location of FSHR on mouse synovium, and found that FSHR is widely expressed on mouse synovium (Fig. 5A). In order to simulate the effect of FSH on OA in perimenopausal women, ovariectomy mice were supplemented with estrogen and treated with FSH. The serum results showed that the OVX + E2 + FSH group had no difference in estrogen and had a higher FSH level compared with the sham group, and the OVX group had lower estrogen (Fig. 5B), indicating the success of animal modeling. H&E staining displayed the increased amount of synovium inflammatory cells in the OVX + E2 + FSH and OVX groups than in the sham group (Fig. 5C). We further labeled macrophages with F4/80 immunostaining, and found that synovial macrophages were significantly increased in the OVX + E2 + FSH and OVX groups than in the sham group (Fig. 5D). Then we used TNF‐α immunostaining to observe the effect of FSH on TNF‐α changes, and the results indicated that TNF‐α in the OVX + E2 + FSH and OVX groups were significantly increased too (Fig. 5E). To investigate whether the changes in the synovial membrane were directly mediated by FSH, we adopted the FSHAb mouse model. Safranin O staining suggested that the damage to cartilage was ameliorated in the FSHAb group (Fig. 5F), indirectly indicating that FSH acted as an adverse factor in OA; then we further observed the changes of synovium and found the inflammatory cells were also decreased in the FSHAb group (Fig. 5G). Above all, the results suggested that FSH activated macrophages in the synovial membrane of the joint.

## Discussion

In our study, FSHR was first shown to be expressed on synovial tissue and macrophages. Previous studies have focused only on the role of FSH on cartilage and the extrachondral matrix [10, 24], but not on the synovium (an independent risk factor of OA). Our study reveals for the first time that FSH‐acting receptor FSHR is also present in synovial tissues and synovial macrophages, indicating the underlying inflammatory role of FSH in synovial tissue.

TNF‐α and other proinflammatory cytokines increased after FSH stimulation in macrophages, and blocking FSHR eliminated this phenomenon, suggesting that FSH acted on macrophages as an inflammation‐promoting mediator and selective inhibition of FSHR in macrophages may be a promising target for the treatment of OA. It is well known that macrophages are the main cells involved in synovial inflammation, and macrophages are activated to release inflammatory cytokines, especially TNF‐α, which triggers synovial inflammation and leads to the progression of OA [25]. TNF‐α is an important mediator of the disturbed processes implicated in OA pathophysiology, which controls the degeneration of the articular cartilage matrix, depletion of macrophages, and neutralization of macrophage‐derived TNF‐α can downregulate matrix metalloproteinase (MMP) production to protect cartilage [26], TNF‐α is also responsible for increased synthesis of IL‐6 and RANTES [27, 28]. In addition to OA, TNF‐α also plays an important role in RA (rheumatic arthritis). RA is an autoimmune disease that has a pathologic basis in synovitis and may eventually lead to joint deformity, which is also prevalent in perimenopausal women. Excessive production of TNF is associated with the development of RA, and biological therapy with TNF inhibitors has radically changed the treatment of rheumatoid arthritis, and is an integral part in the management [29, 30].

The aggregation of synovial macrophages and the increase of TNF‐α in the joints of mice in the higher FSH group (OVX, OVX + E2 + FSH) was found in our research, which is consistent with the *in vitro* results, suggesting activation of synovial inflammation. The ovariectomized model was widely used to stimulate postmenopausal women who displayed a high dose of FSH and low dose of estrogen, so we used the OVX model as a positive control group. In order to simulate the effect of FSH on OA in perimenopausal women, we supplemented OVX mice with estrogen chow to exclude an estrogenic effect and treated them with FSH, which has been widely used in animal studies related to OA [10, 31].

In order to investigate the direct effect of FSH deficiency on synovial membrane of the joint, we used the FSHAb model, which has been validated in several experiments [10, 18]., Interestingly, blocking FSH alleviated the inflammatory response of the synovial membrane. Our results indicated that FSH had an independent inflammatory effect on the synovial membrane, which was consistent with our cell results.

Our previous study suggested the NFκB pathway was activated in cartilage after FSH stimulation; therefore, it is likely to be the pathway through which FSH exerts its proinflammatory influence on macrophages. Our results were consistent with the speculation that FSH stimulation activated the NFκB signaling pathway, and inhibition of FSH alleviated this phenomenon, indicating that FSH might exert its proinflammatory influence through activating the NFκB signaling pathway. The NFκB signaling pathway is involved in many OA events, such as chondrocyte breakdown, extrachondral matrix (ECM) degradation, and synovial inflammation [17, 32, 33]. When cells are induced by inflammatory mediators, the NFκB inhibitor IKB is phosphorylated by IκB kinaseβ (IKKβ) and dissociated, thus leading to P65 phosphorylation and exposure of the NFκB dimer (P65/P50). At this time, the P65/P50 enters the nucleus to stimulate DNA transcription and then promotes the expression of inflammatory cytokines, adhesion molecules, and growth factors so as to regulate the physiological and pathological processes of tissues and cells [34].

As is known to all, the pathogenesis of OA is not clear at present, and there are no drugs to interfere with the progression of OA. The treatment measures for OA are mainly drugs to relieve symptoms. So, this brings challenges for the early prevention and treatment of OA. Therefore, we further explored the pathogenesis of OA regulated by FSH, which has important scientific significance for exploring effective intervention targets for the early prevention and treatment of OA.

There are some limitations in our present study. First, we did not study the effect of FSH on primary synovial macrophages due to the unavailability of methods for extracting synovial macrophages. Second, we did not explore the interaction between macrophages and chondrocytes, which facilitate a more specific and in‐depth elucidation of the mechanisms of FSH action on OA. Third, there is a feedback axis between sex hormones such as FSH/LN, estrogen, and testosterone, which affect each other and their sensitivities, and the experimental design of the animals in this study may be interfered by other sex hormones, such as testosterone. Last, some of our experiments may not be fully powered. We will continue to conduct experiments to verify our conclusions if needed. In addition, FSH synovial‐specific knockout animal models are also needed to access a direct effect of FSH on synovial macrophages in OA.

In conclusion, our study was the first to demonstrate that FSHR was expressed in synovial macrophages and that FSH played a proinflammatory role in synovial macrophages, possibly by activating the NFκB pathway to be involved in the pathogenesis of postmenopausal osteoarthritis. These results may, to some extent, elucidate the cellular and molecular mechanisms of postmenopausal OA. Our study may provide some insights and ideas for the prevention and treatment of osteoarthritis in postmenopausal women.

## Conflict of interest

The authors declare no conflict of interest.

### Peer review

The peer review history for this article is available at https://www.webofscience.com/api/gateway/wos/peer‐review/10.1002/2211‐5463.13959.

## Author contributions

YC, Y‐mZ, and JX designed the study and prepared the first draft of the paper. W‐wZ, NX, and YW contributed to the experimental work. TS was responsible for statistical analysis of the data. All authors revised the paper critically for intellectual content and approved the final version. All authors agree to be accountable for the work and to ensure that any questions relating to the accuracy and integrity of the paper are investigated and properly resolved.

## Supporting information


**Fig. S1.** Mean fluorescence intensity of different cytokines in macrophages.
**Fig. S2.** Statistical comparison of Luminex data.
**Table S1.** Primer's sequence used for qRT‐PCR.
**Table S2.** Primer sequences of FSHR siRNA.

## Data Availability

The data that support the findings of this study are available from the corresponding author xujin@sdfmu.edu.cn upon reasonable request.

## References

[1] Loeser RF (2006) Molecular mechanisms of cartilage destruction: mechanics, inflammatory mediators, and aging collide. Arthritis Rheum 54, 1357–1360. 16645963 10.1002/art.21813 PMC1774815

[2] Berenbaum F (2013) Osteoarthritis as an inflammatory disease (osteoarthritis is not osteoarthrosis!). Osteoarthr Cartil 21, 16–21. 10.1016/j.joca.2012.11.012 23194896

[3] Sellam J and Berenbaum F (2010) The role of synovitis in pathophysiology and clinical symptoms of osteoarthritis. Nat Rev Rheumatol 6, 625–635. 20924410 10.1038/nrrheum.2010.159

[4] Felson DT , Niu J , Neogi T , Goggins J , Nevitt MC , Roemer F , Torner J , Lewis CE , Guermazi A and MOST Investigators Group (2016) Synovitis and the risk of knee osteoarthritis: the MOST study. Osteoarthr Cartil 24, 458–464. 10.1016/j.joca.2015.09.013 PMC4761323 26432512

[5] Xie F , Kovic B , Jin X , He X , Wang M and Silvestre C (2016) Economic and humanistic burden of osteoarthritis: a systematic review of large sample studies. Pharmacoeconomics 34, 1087–1100. 27339668 10.1007/s40273-016-0424-x

[6] World Health Organization (2016) China national aging and health assessment report[R]. WHO, Geneva, Switzerland:12–22

[7] Taneja C , Gera S , Kim SM , Iqbal J , Yuen T and Zaidi M (2019) FSH‐metabolic circuitry and menopause. J Mol Endocrinol 63, R73–R80. 31454787 10.1530/JME-19-0152 PMC6992500

[8] Liu Y , Zhang M , Kong D , Wang Y , Li J , Liu W , Fu Y and Xu J (2020) High follicle‐stimulating hormone levels accelerate cartilage damage of knee osteoarthritis in postmenopausal women through the PI3K/AKT/NF‐κB pathway. FEBS Open Bio 10, 2235–2245. 10.1002/2211-5463.12975 PMC7530390 32911565

[9] Siraj A , Desestret V , Antoine M , Fromont G , Huerre M , Sanson M , Camparo P , Pichon C , Planeix F , Gonin J *et al*. (2013) Expression of follicle‐stimulating hormone receptor by the vascular endothelium in tumor metastases. BMC Cancer 13, 246. 23688201 10.1186/1471-2407-13-246 PMC3663659

[10] Wang Y , Zhang M , Huan Z , Shao S , Zhang X , Kong D and Xu J (2021) FSH directly regulates chondrocyte dedifferentiation and cartilage development. J Endocrinol 248, 193–206. 33295881 10.1530/JOE-20-0390

[11] Robinson LJ , Tourkova I , Wang Y , Sharrow AC , Landau MS , Yaroslavskiy BB , Sun L , Zaidi M and Blair HC (2010) FSH‐receptor isoforms and FSH‐dependent gene transcription in human monocytes and osteoclasts. Biochem Biophys Res Commun 394, 12–17. 20171950 10.1016/j.bbrc.2010.02.112 PMC2856932

[12] Scanzello CR and Goldring SR (2012) The role of synovitis in osteoarthritis pathogenesis. Bone 51, 249–257. 22387238 10.1016/j.bone.2012.02.012 PMC3372675

[13] Mathiessen A and Conaghan PG (2017) Synovitis in osteoarthritis: current understanding with therapeutic implications. Arthritis Res Ther 19, 18. 28148295 10.1186/s13075-017-1229-9 PMC5289060

[14] Klein‐Wieringa IR , de Lange‐Brokaar BJ , Yusuf E , Andersen SN , Kwekkeboom JC , Kroon HM , van Osch GJVM , Zuurmond A‐M , Stojanovic‐Susulic V , Nelissen RGHH *et al*. (2016) Inflammatory cells in patients with Endstage knee osteoarthritis: a comparison between the synovium and the infrapatellar fat pad. J Rheumatol 43, 771–778. 26980579 10.3899/jrheum.151068

[15] Goldring MB (2012) Chondrogenesis, chondrocyte differentiation, and articular cartilage metabolism in health and osteoarthritis. Ther Adv Musculoskelet Dis 4, 269–285. 22859926 10.1177/1759720X12448454 PMC3403254

[16] Yang L , Chen Z , Guo H , Wang Z , Sun K , Yang X , Zhao X , Ma L , Wang J , Meng Z *et al*. (2021) Extensive cytokine analysis in synovial fluid of osteoarthritis patients. Cytokine 143, 155546. 33895075 10.1016/j.cyto.2021.155546

[17] Xiao L , Zhong M , Huang Y , Zhu J , Tang W , Li D , Shi J , Lu A , Yang H , Geng D *et al*. (2020) Puerarin alleviates osteoporosis in the ovariectomy‐induced mice by suppressing osteoclastogenesis via inhibition of TRAF6/ROS‐dependent MAPK/NF‐κB signaling pathways. Aging 12, 21706–21729. 33176281 10.18632/aging.103976 PMC7695364

[18] Guo Y , Zhao M , Bo T , Ma S , Yuan Z , Chen W , He Z , Hou X , Liu J , Zhang Z *et al*. (2019) Blocking FSH inhibits hepatic cholesterol biosynthesis and reduces serum cholesterol. Cell Res 29, 151–166. 30559440 10.1038/s41422-018-0123-6 PMC6355920

[19] Li D , Ruan G , Zhang Y , Zhao Y , Zhu Z , Ou Q , Huang H , Chen J , Han W , Tang S'a *et al*. (2022) Metformin attenuates osteoarthritis by targeting chondrocytes, synovial macrophages and adipocytes. Rheumatology (Oxford) 62, 1652–1661. 10.1093/rheumatology/keac467 35984286

[20] Wang D , Wu Z , Zhao C , Yang X , Wei H , Liu M , Zhao J , Qian M , Li Z and Xiao J (2021) KP‐10/Gpr54 attenuates rheumatic arthritis through inactivating NF‐κB and MAPK signaling in macrophages. Pharmacol Res 171, 105496. 33609696 10.1016/j.phrs.2021.105496

[21] Baker RG , Hayden MS and Ghosh S (2011) NF‐κB, inflammation, and metabolic disease. Cell Metab 13, 11–22. 21195345 10.1016/j.cmet.2010.12.008 PMC3040418

[22] Jimi E , Fei H and Nakatomi C (2019) NF‐κB signaling regulates physiological and pathological chondrogenesis. Int J Mol Sci 20, 6275. 31842396 10.3390/ijms20246275 PMC6941088

[23] Sueishi T , Akasaki Y , Goto N , Kurakazu I , Toya M , Kuwahara M , Uchida T , Hayashida M , Tsushima H , Bekki H *et al*. (2020) GRK5 inhibition attenuates cartilage degradation via decreased NF‐κB signaling. Arthritis Rheumatol 72, 620–631. 31696655 10.1002/art.41152

[24] Zhang M , Wang Y , Huan Z , Liu Y , Zhang W , Kong D , Kong L and Xu J (2021) FSH modulated cartilage ECM metabolism by targeting the PKA/CREB/SOX9 pathway. J Bone Miner Metab 39, 769–779. 33988757 10.1007/s00774-021-01232-3

[25] Kapoor M , Martel‐Pelletier J , Lajeunesse D , Pelletier JP and Fahmi H (2011) Role of proinflammatory cytokines in the pathophysiology of osteoarthritis. Nat Rev Rheumatol 7, 33–42. 21119608 10.1038/nrrheum.2010.196

[26] Liu P , Ji Y , Yuen T , Rendina‐Ruedy E , DeMambro VE , Dhawan S , Abu‐Amer W , Izadmehr S , Zhou B , Shin AC *et al*. (2017) Blocking FSH induces thermogenic adipose tissue and reduces body fat. Nature 546, 107–112. 28538730 10.1038/nature22342 PMC5651981

[27] Guerne PA , Carson DA and Lotz M (1990) IL‐6 production by human articular chondrocytes. Modulation of its synthesis by cytokines, growth factors, and hormones in vitro. J Immunol 144, 499–505. 2104896

[28] Alaaeddine N , Olee T , Hashimoto S , Creighton‐Achermann L and Lotz M (2001) Production of the chemokine RANTES by articular chondrocytes and role in cartilage degradation. Arthritis Rheum 44, 1633–1643. 11465714 10.1002/1529-0131(200107)44:7<1633::AID-ART286>3.0.CO;2-Z

[29] Zhai Z , Yang F , Xu W , Han J , Luo G , Li Y , Zhuang J , Jie H , Li X , Shi X *et al*. (2022) Attenuation of rheumatoid arthritis through the inhibition of tumor necrosis factor‐induced caspase 3/Gasdermin E‐mediated Pyroptosis. Arthritis Rheum 74, 427–440. 10.1002/art.41963 PMC9305212 34480835

[30] Chaabo K and Kirkham B (2015) Rheumatoid arthritis – anti‐TNF. Int Immunopharmacol 27, 180–184. 25962818 10.1016/j.intimp.2015.04.051

[31] Chazaud B (2020) Inflammation and skeletal muscle regeneration: leave it to the macrophages! Trends Immunol 41, 481–492. 32362490 10.1016/j.it.2020.04.006

[32] Yan H , Duan X , Pan H , Holguin N , Rai MF , Akk A , Springer LE , Wickline SA , Sandell LJ and Pham CT (2016) Suppression of NF‐κB activity via nanoparticle‐based siRNA delivery alters early cartilage responses to injury. Proc Natl Acad Sci U S A 113, E6199–E6208. 27681622 10.1073/pnas.1608245113 PMC5068304

[33] Marcu KB , Otero M , Olivotto E , Borzi RM and Goldring MB (2010) NF‐kappaB signaling: multiple angles to target OA. Curr Drug Targets 11, 599–613. 20199390 10.2174/138945010791011938 PMC3076145

[34] Choi MC , Jo J , Park J , Kang HK and Park Y (2019) NF‐κB signaling pathways in osteoarthritic cartilage destruction. Cells 8, 734. 31319599 10.3390/cells8070734 PMC6678954

