# Supplementary material for: FSH enhances the inflammatory response of macrophages in the knee joint possibly through the NFκB pathway
Source: FEBS Open Bio. 2025 Jan 13;15(4):622–33. doi: 10.1002/2211-5463.13959 (PMC11961395; doi:10.1002/2211-5463.13959)
Supplement: Supplementary file 1 — Fig. S1. Mean fluorescence intensity of different cytokines in macrophages. Fig. S2. Statistical comparison of Luminex data. Table S1. Primer's sequence used for qRT‐PCR. Table S2. Primer sequences of FSHR siRNA. [file FEB4-15-622-s001.docx]

Supplementary tables:

Supplementary table1:

Table1. primers sequence used for qRT-PCR

| Gene name | Forward and reverse primer (5’–3’) |
| --- | --- |
| Actin | F:GGCTGTATTCCCCTCCATCG R:CCAGTTGGTAACAATGCCATGT |
| FSHR | F:CCTTGCTCCTGGTCTCCTTG  R:CTCGGTCACCTTGCTATCTTG |
| TNF-α | F:CATCTTCTCAAAATTCGAGTGACAA R:TGGGAGTAGACAAGGTACAACCC |
| IL-6 | F:TAGTCCTTCCTACCCCAATTTCC R:TTGGTCCTTAGCCACTCCTTC |
| IL-10 | F:GCTCTTACTGACTGGCATGAG R:CGCAGCTCTAGGAGCATGTG |

Supplementary table2:

Table 2 primer sequences of FSHR siRNA

| Gene name | Forward and reverse primer (5’–3’) |
| --- | --- |
| si-1# | F:GAACUGAAUCUAAGCGAUATT R:UAUCGCUUAGAUUCAGUUCTT |
| si-2# | F:AGCCAUAACCCUAGAAAGATT R:UCUUUCUAGGGUUAUGGCUTT |
| si-3# | F:CAGAUUUACAAGACAGAAATT R:UUUCUGUCUUGUAAAUCUGTT |

Supplementary figures


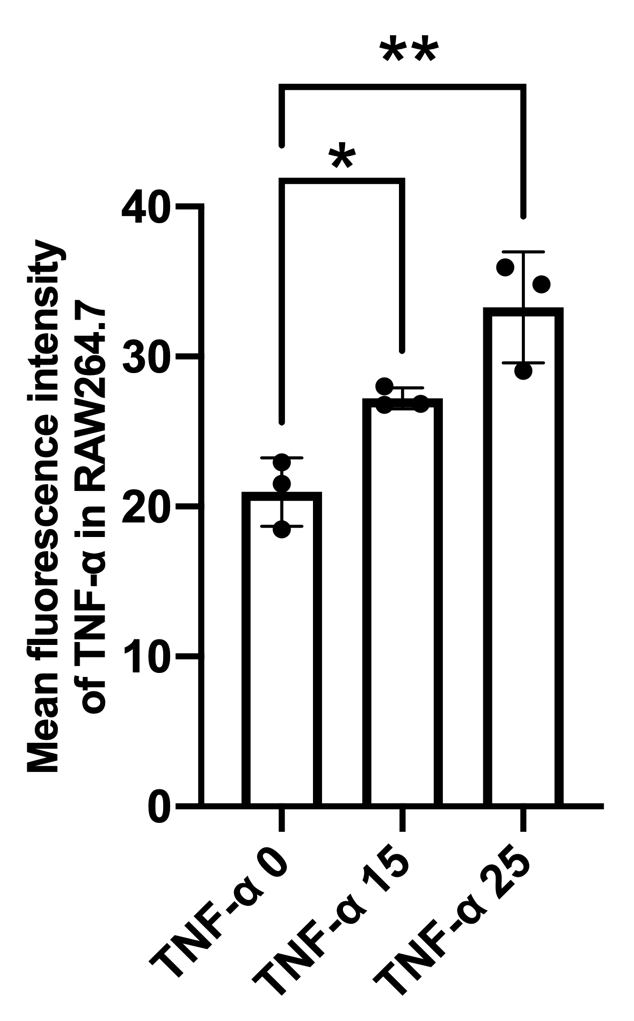

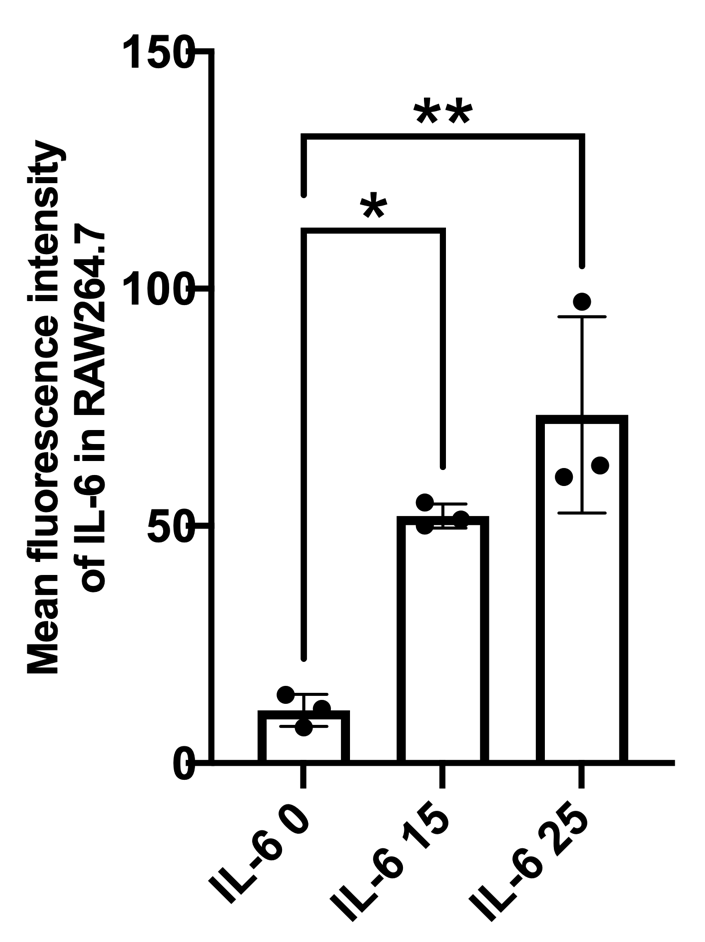


Fig S1: Mean fluorescence intensity of different cytokines in macrophages, (A)Mean fluorescence intensity of TNF-α in RAW264.7 cell line, n=3, data are presented as means ± SD, data were analyzed by One‐way ANOVA, *P<0.05,**P<0.01. (B)Mean fluorescence intensity of IL-6 in RAW264.7 cell line, n=3, data are presented as means ± SD, data were analyzed by One‐way ANOVA, *P<0.05,**P<0.01.


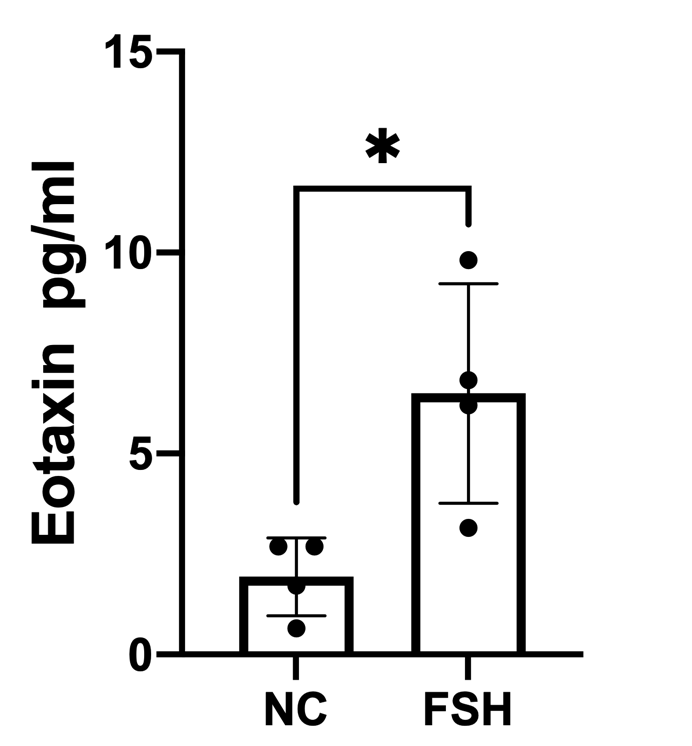

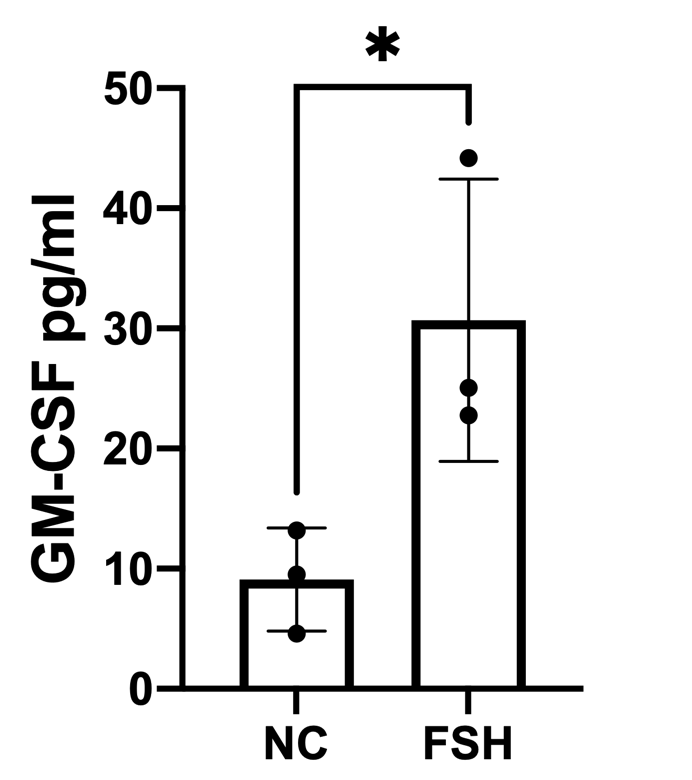

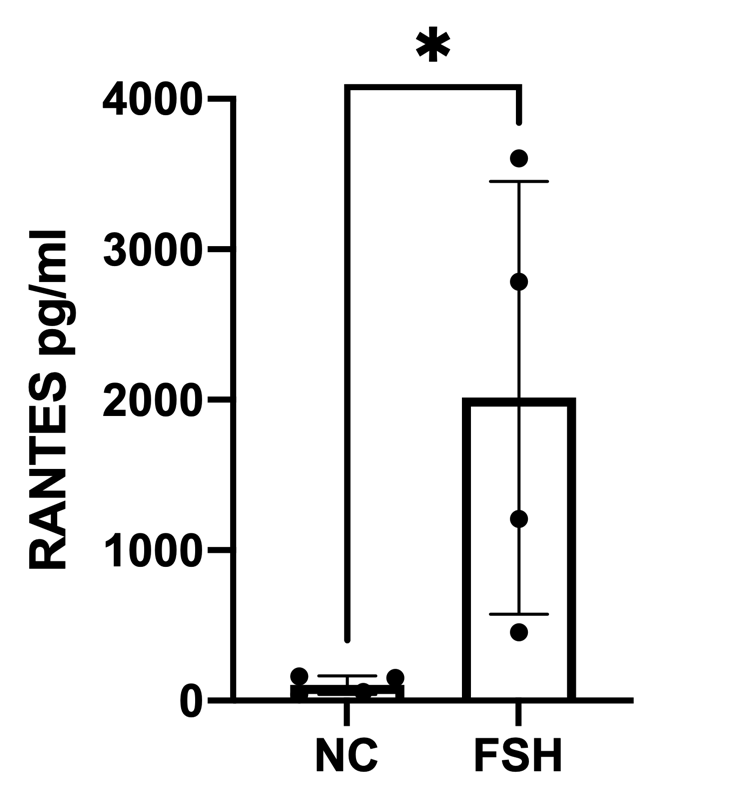

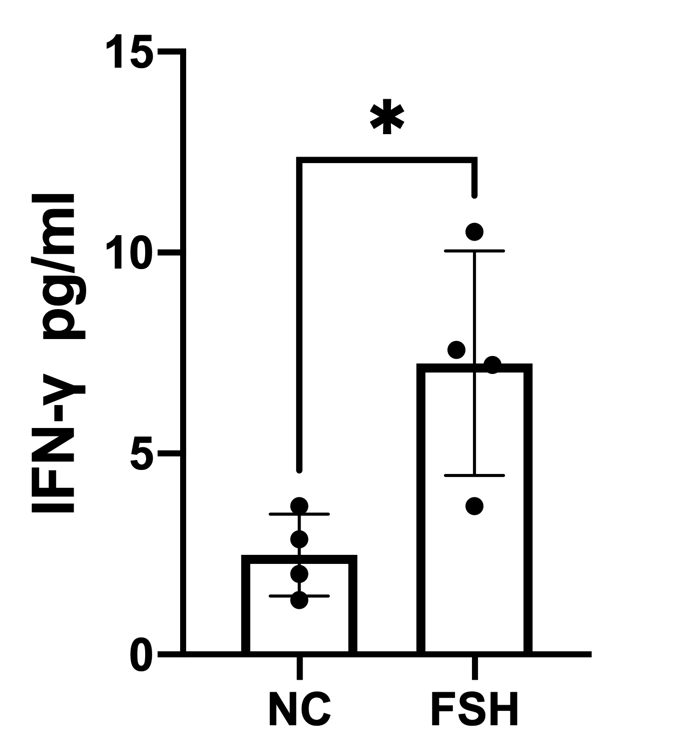


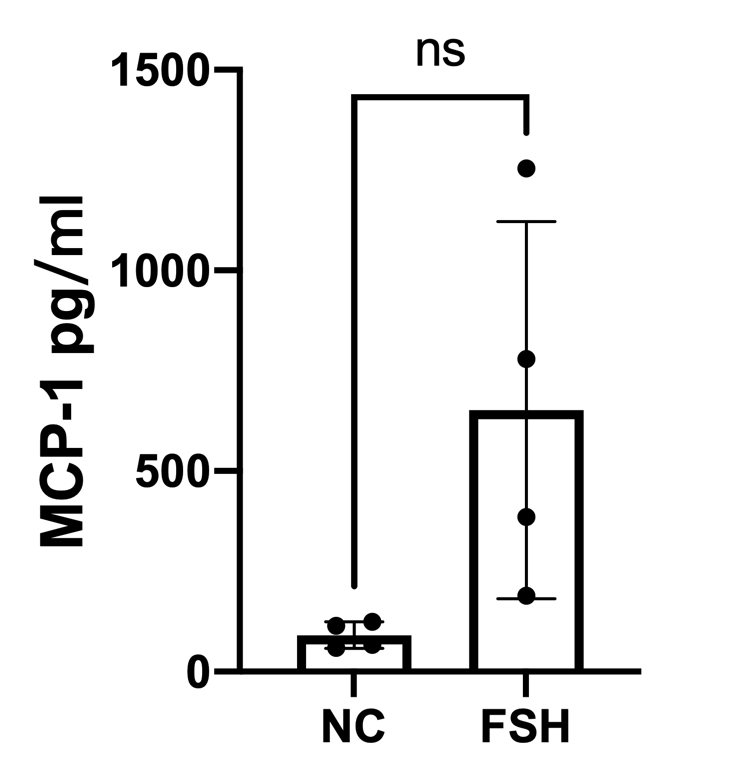

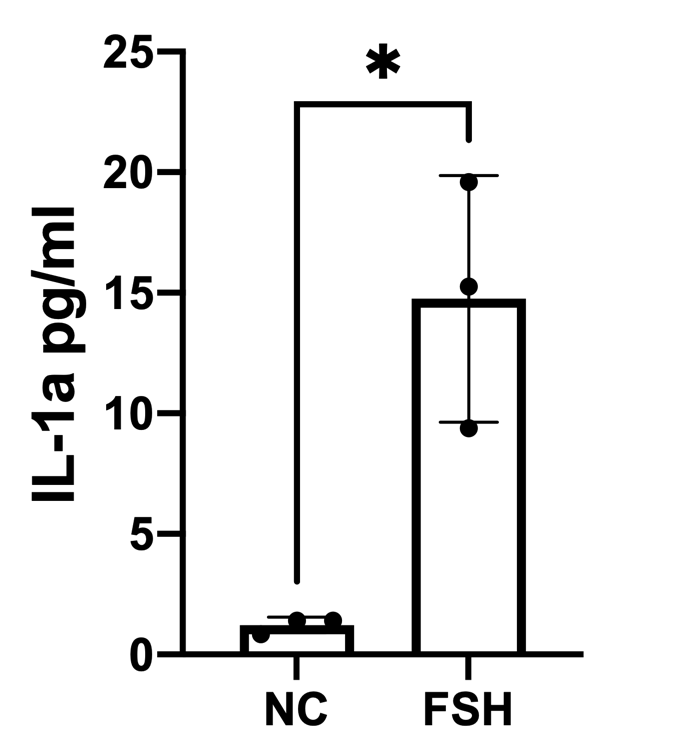


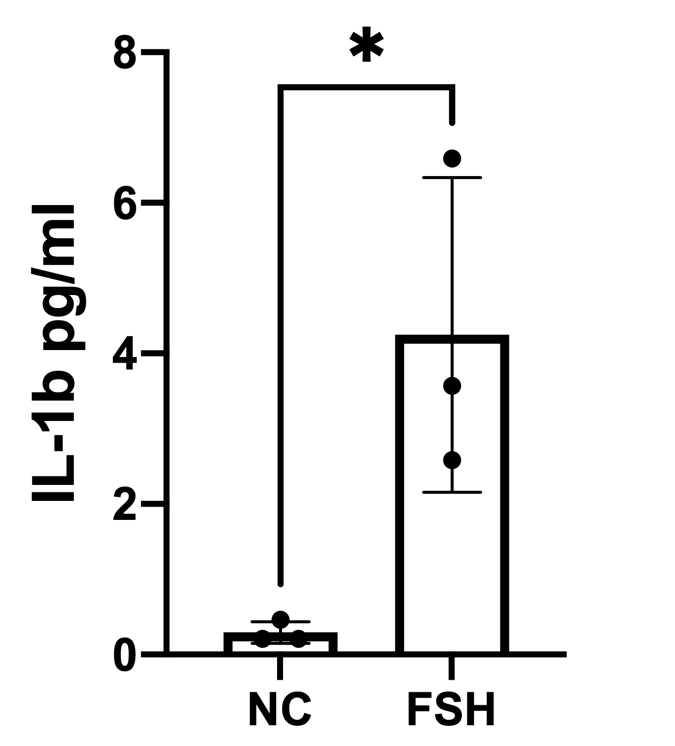

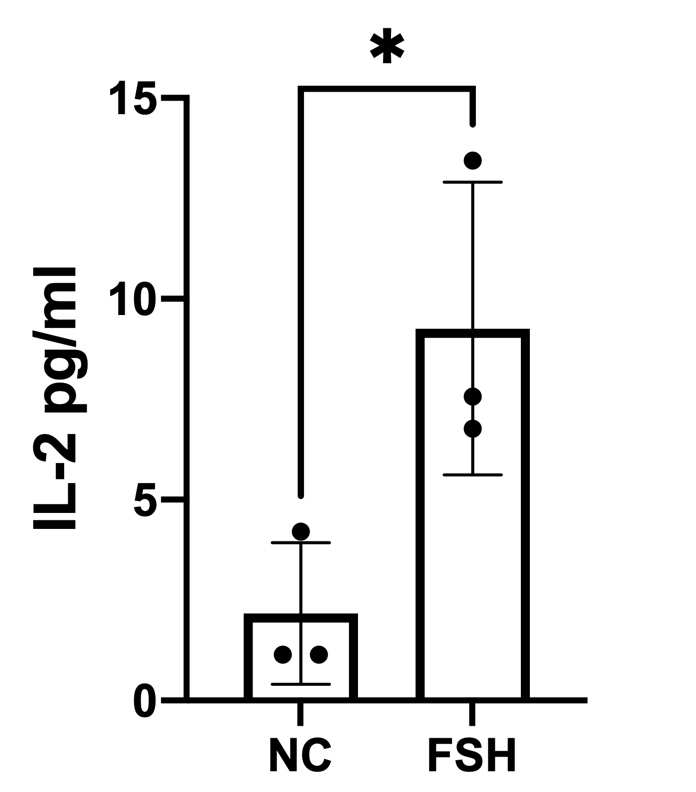


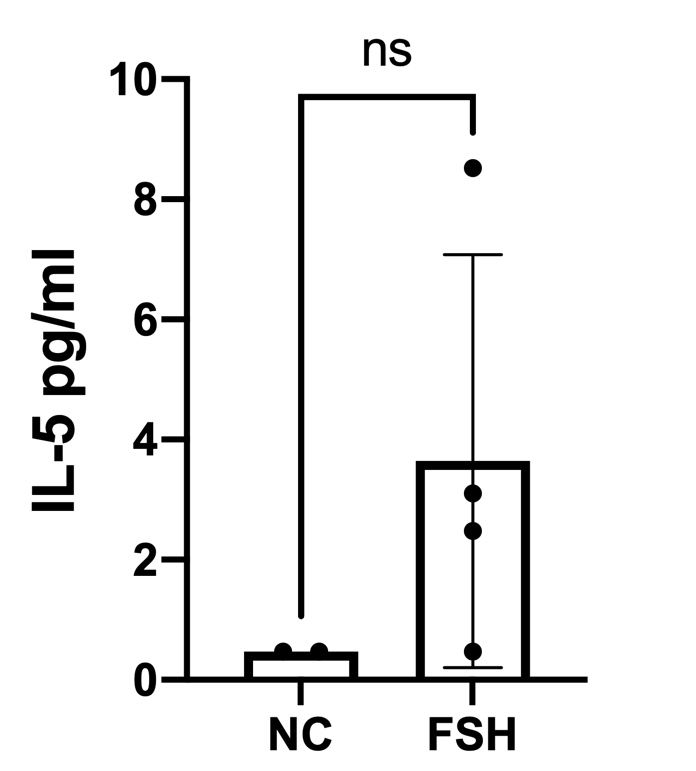

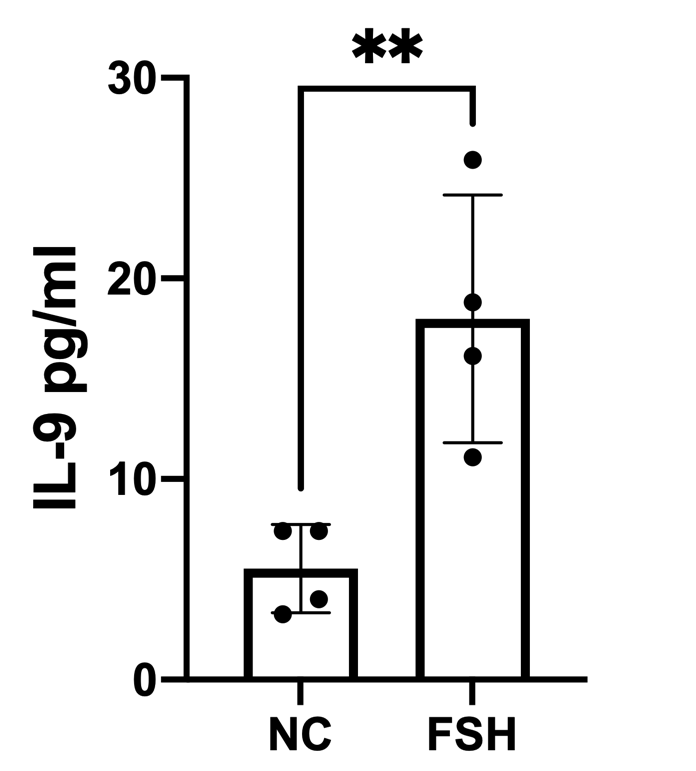


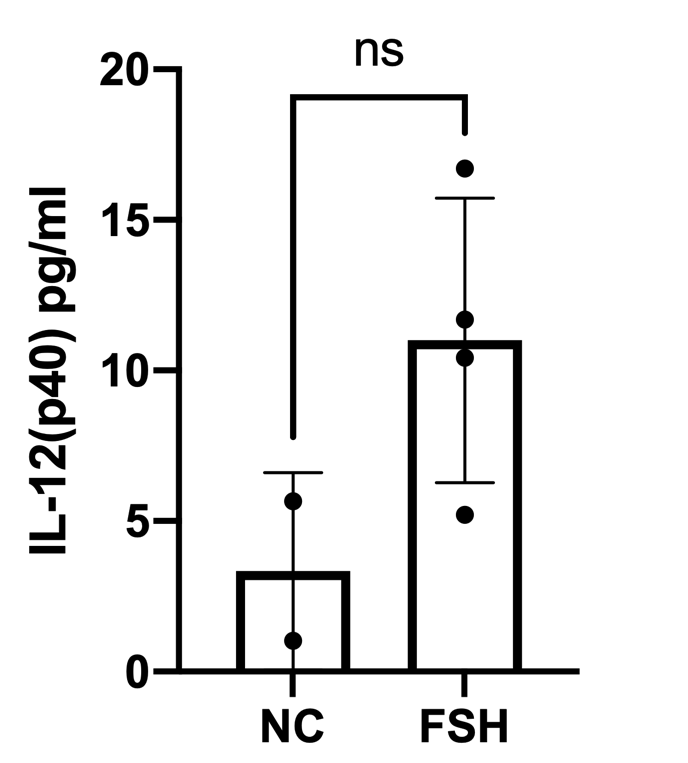

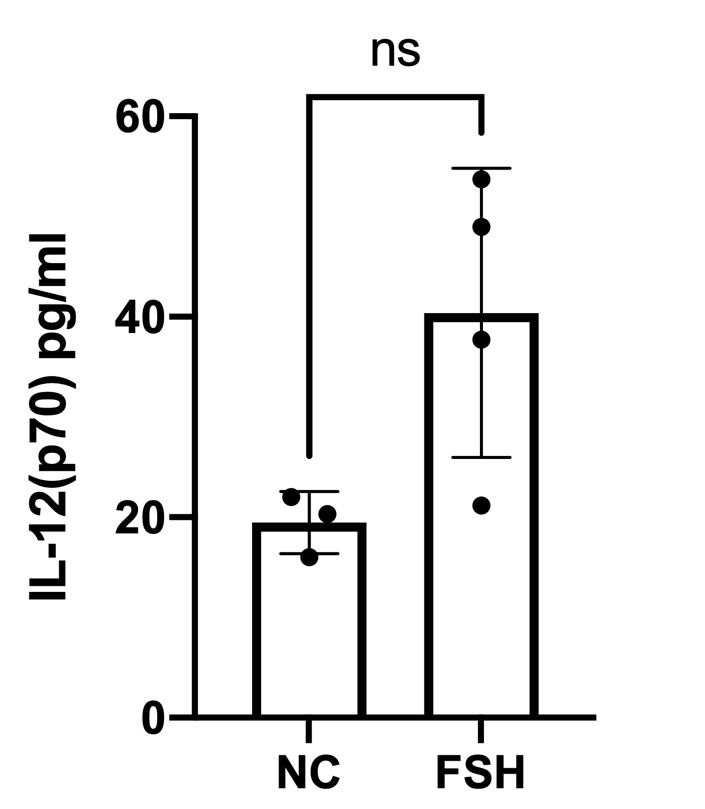


FigS2: Statistical comparison of Luminex data. (A-L) Inflammatory cytokines concentrations in cell culture supernatant of NC and 25ng/ml FSH groups were detected by Luminex, n=4 (A-E, I-L), n=3 (F-H), data are presented as means ± SD, data were analyzed by T-test, * P<0.05, ** P< 0.001 relative to NC.
